# European Society of Urogenital Radiology (ESUR) Guidelines: MR Imaging of Leiomyomas

**DOI:** 10.1007/s00330-017-5157-5

**Published:** 2018-02-28

**Authors:** Rahel A. Kubik-Huch, Michael Weston, Stephanie Nougaret, Henrik Leonhardt, Isabelle Thomassin-Naggara, Mariana Horta, Teresa Margarida Cunha, Cristina Maciel, Andrea Rockall, Rosemarie Forstner

**Affiliations:** 1 Institut für Radiologie, Kantonsspital Baden AG, CH-5404 Baden-Dättwil, Switzerland; 2 Department of Radiology, Leeds Teaching Hospitals NHS Trust, Leeds, UK; 3 IRCM, Montpellier Cancer Research institute, 208 Ave des Apothicaires, Montpellier, 34295 France; 4 Department of Radiology, Montpellier Cancer Institute INSERM, U1194, University of Montpellier, 208 Ave des Apothicaires, Montpellier, 34295 France; 5 Överläkare, med dr. Radiologi Buk/Kärl-sektionen, Sahlgrenska Universitetssjukhuset-S, Bruna stråket 11B, 413 45 Göteborg, Sweden; 6 Hôpital Tenon, 4 rue de la Chine, F- 75020 Paris, France; 7 Departament of Radiology, Instituto Português de Oncologia de Lisboa Francisco Gentil, R. Prof. Lima Basto, 1099-023 Lisbon, Portugal; 8 Radiology Department, Hospital São João, Alameda Prof. Hernâni Monteiro, 4200–319 Porto, Portugal; 9 Department of Radiology, The Royal Marsden NHS Foundation Trust, London, UK; 10 Division of Surgery and Cancer, Faculty of Medicine, Imperial College London, London, UK; 11 Department of Radiology, Universitätsklinikum Salzburg, PMU; Müllner Hauptstr. 48, A-5020 Salzburg, Austria

**Keywords:** Leiomyoma, Uterus, Magnetic resonance imaging, Genital diseases female, Guideline

## Abstract

**Objective:**

The aim of the Female Pelvic Imaging Working Group of the European Society of Urogenital Radiology (ESUR) was to develop imaging guidelines for MR work-up in patients with known or suspected uterine leiomyomas.

**Methods:**

Guidelines for imaging uterine leiomyomas were defined based on a survey distributed to all members of the working group, an expert consensus meeting at European Congress of Radiology (ECR) 2017 and a critical review of the literature.

**Results:**

The 25 returned questionnaires as well as the expert consensus meeting have shown reasonable homogeneity of practice among institutions. Expert consensus and literature review lead to an optimized MRI protocol to image uterine leiomyomas. Recommendations include indications for imaging, patient preparation, MR protocols and reporting criteria. The incremental value of functional imaging (DWI, DCE) is highlighted and the role of MR angiography discussed.

**Conclusions:**

MRI offers an outstanding and reproducible map of the size, site and distribution of leiomyomas. A standardised imaging protocol and method of reporting ensures that the salient features are recognised. These imaging guidelines are based on the current practice among expert radiologists in the field of female pelvic imaging and also incorporate essentials of the current published MR literature of uterine leiomyomas.

***Key Points*:**

• *MRI allows comprehensive mapping of size and distribution of leiomyomas.*

• *Basic MRI comprise T2W and T1W sequences centered to the uterus.*

• *Standardized reporting ensures pivotal information on leiomyomas, the uterus and differential diagnosis.*

• *MRI aids in differentiation of leiomyomas from other benign and malignant entities, including leiomyosarcoma.*

**Electronic supplementary material:**

The online version of this article (10.1007/s00330-017-5157-5) contains supplementary material, which is available to authorized users.

## Introduction

Leiomyomas (syn: myomas or fibroids) constitute the most common benign uterine tumours. They are typically multiple and are classified as submucosal, intramural and subserosal. It is estimated that 20-50% become symptomatic, with symptoms including abnormal uterine bleeding, pelvic pain, bowel or urinary obstruction, or pregnancy related complications [1]. Due to their hormonal sensitivity, they shrink in menopause and occasionally may rapidly grow and bleed during pregnancy. Torsion or infarction can cause an acute abdomen [2].

Sonography is the first line imaging modality to assess leiomyomas. MRI, due to its better soft tissue contrast, larger field-of-view and multiplanar imaging capabilities, assists clinicians in complementary pre-treatment mapping (exact origin, number and size) and in differential diagnostic issues [3]. MRI is better able than ultrasound to distinguish malignant ovarian lesions that might otherwise mimic leiomyomas. MRI is also better able to detect features of malignant change in a leiomyoma, which although rare, are of vital importance if the leiomyoma is being considered for morcellation during laparoscopic removal. Morcellation carries the risk of disseminating malignant tissue. MRI has been shown to impact on treatment planning in about 20 % of patients with symptomatic leiomyomas [4–6]. Although not mandatory with high-quality ultrasonography, MRI has become the mainstay of diagnosis and has an important role for treatment planning and monitoring of uterine embolization.

Clinical guidelines for diagnostic work-up and treatment of patients with leiomyomas are well established (e.g. NICE clinical knowledge summary, Fibroids 2013. cks.nice.org.uk and the Society of Obstetricians and Gynaecologists of Canada, clinical practice guideline 318, February 2015). However, despite its wide utilisation, a lack of standardised recommendations/guidelines for MR imaging and reporting of leiomyomas is notable.

These recommendations prepared by the Female Pelvic Imaging Working Group of the European Society of Urogenital Radiology (ESUR) are based on the current practice among specialist radiologists in the field of female pelvic imaging, and also incorporate essentials in MRI in the light of the current published literature of uterine leiomyomas.

## Materials and methods

### Questionnaire

A questionnaire was designed (RKH) and then amended by two other authors (MW, RF). Indications, clinical practice, technical details including patient preparation, the MRI protocol and details of reporting were assessed. The questionnaire is added in the supplementary material. For some questions more than one answer was possible. Not all answers were ticked by all respondents.

Institutional Review Board approval was not required because this manuscript presents guidelines only, without the use of patient-sensitive data.

The questionnaire was distributed to all members (55 members from 46 different institutions) of the Female Pelvic Imaging Working Group of the ESUR. All surveys returned by January 15^th^ 2017 were included in the analysis.

### Literature search

In addition, published literature between 2007 and 2017 from Medline (Ovid), EMBASE (embase.com) and Cochrane Library was reviewed. The literature was searched with the concepts of MRI and Uterine Leiomyoma and its synonyms (angioleiomyoma* or angiomyoma* or elastomyofibroma* or fibromyoma* or hemangioleiomyoma* or hemangiomyoma* or leiomyoma* or myofibroma* or myofibromatos* or fibroid* or fibromyoma* or myoma* in combination with uterine or uterus), using a combination of MeSH- respectively EMTREE-Terms and freetext search in the title and abstract. The search includes original research articles as well as review articles. The results were limited to English language and to publications from important radiology and gynaecology journals (impact factor above 1).

### Consensus meeting

Results were discussed at the Female Imaging Working Group meeting at the ECR 2017 on March 2, 2017 in Vienna and recommendations amended based on the inputs of the members present. A draft manuscript proposing an imaging algorithm for the work-up of uterine leiomyoma was then developed by the three leading authors (RKH, MW, RF) and subsequently finalized with contributions from the other co-authors. The final version of the manuscript was circulated among members of the Female Pelvic Imaging Subcommittee for approval.

## Results

### Participating institutions

Most of the 26 questionnaires submitted were from European countries:

- Portugal: n=5
- France: n= 3
- Croatia: n=1
- Spain: n=1
- United Kingdom: n= 4
- Switzerland: n=3
- Germany: n=1
- Austria: n=2
- Sweden: n=1
- Italy: n=1
- Greece: n=1
- Serbia: n=1

Furthermore, a questionnaire was submitted from Kyoto, Japan as well as from New York, USA.

Two questionnaires were submitted from the same institution; with the one being submitted later excluded from analysis. Thus, 25 surveys from different institutions were available for analysis. The return rate on an institutional level was therefore 54.3%.

The approximate number of *MR exams per year* for assessing uterine leiomyomas was reported by each institution:

- more than 50 exams: n=10
- 20-50 exams: n=11
- 10-20 exams: n=3
- less than 10 exams: n=1

The *following treatment* options are offered to the patients at the 25 participating institutions:

- open surgery (hysterectomy, myomectomy): n= 24
- laparoscopic surgery: n=24
- medical/hormone therapy: n=22
- embolization of uterine leiomyomas: n=14
- Magnetic resonance guided focused ultrasound (MRgFUS): n=1

*Indications for MR imaging of leiomyomas*:

All (n=25) use MR to differentiate leiomyomas from indeterminate adnexal masses or in indeterminate ultrasound. Other common indications are listed below:

- assessment of pelvic pain or other clinical symptoms: n=22
- differential diagnosis of leiomyosarcoma: n=22
- treatment planning in symptomatic patients: n=22
- imaging of infertility: n=16
- therapy monitoring after treatment: n=16

Previous ultrasound examination of the uterus is performed by gynaecologists (n=21) and/or radiologists (n=16) and/or sonographers (n=2). When reporting MR scans, ultrasound images are available always or mostly in 13 centres, while images can be used for comparison only sometimes or not at all in the other institutions.

### Role of computed tomography

Twenty respondents did not see any role for computed tomography (CT) in assessing uterine leiomyomas. Five believe CT plays a role in selected (n=4) or all (n=1) indications (e.g. acute pain, to assess calcifications or CT angiography in the follow-up of uterine artery embolization with incomplete response).

### Patient preparation

In most institutions (n=20/25), the patient is not scheduled according to her menstrual cycle, whereas in 4 institutions the menstrual cycle is considered. No answer was given by one institution.

About two-thirds of the participants (n=17/25) ask for clinical symptoms or provide a questionnaire including questions such as time of last menstruation, clinical symptoms or hormonal medication.

Seventeen of 25 sites ask the patient to fast before the examination. Fasting ranges between 2-6 hours, mostly (n=9) of 4 hours.

Only 4 institutions never use antispasmolytic agents. Glucagon® (n= 9) and/or Buscopan® (n= 15) is given in the other 21 institutions. Whereas Glucagon® is administered intravenously, for Buscopan® intravenous and intramuscular applications are used.

There was agreement at the ECR consensus meeting that a “half-full”, moderately distended urinary bladder is optimal for pelvic MR assessment, with the instructions given to the patients to achieve this varying among the institutions.

### MR equipment & MR protocol

Different machines (GE, Philipps, Siemens) are used. Nine institutions performed MRI at 1.5 Tesla only, 3 institutions at 3 Tesla only, in 11 institutions scanners with both field strengths were used; no answer regarding MR equipment was given by 1 institution.

A variety of coils are used for imaging with a preference for pelvic phased array coils and multi-channel body coils.

Patient positioning (head first or feet first) is not uniform; some institutions remarked that this depended on the clinical question.

Imaging of the upper abdomen and kidneys is included by 10/25 sites. All perform a sagittal T2W (weighted) series of the female pelvis and the uterus. Additional oblique T2W images are performed in 17, whereas two centers perform axial and coronal T2W scans without angulations to the uterus.

Twenty-one centers perform axial, native T1W of the pelvis, one group prefers coronal T1W imaging and two others sagittal T1W pre-contrast planes. T1WFS depends on institutional preference and on clinical questions. For distinction of adenomyosis from subserosal endometriosis, precontrast FS T1W sequences are considered most helpful.

Diffusion-weighed imaging was routinely used by 21 members and sometimes by 4 others. The rationale given is that DWI is part of the routine uterine protocol but not used specifically for leiomyomas.

Two institutions never use intravenous Gadolinium for imaging of leiomyoma. All the other institutions use Gadolinium either routinely administered (n=12) or depending on the clinical question (n=11). Indications for intravenous administration include treatment planning, differential diagnosis of leiomyosarcoma, and differential diagnosis from an adnexal mass as well as follow up of uterine artery embolization.

Dynamic contrast enhanced imaging (DCE) is routinely performed in 5 sites and in selected cases in 11 sites, whereas 9 institutions never use dynamic imaging. Indications for DCE include the differential diagnosis from an adnexal mass, differential diagnosis of leiomyosarcoma, or vascularisation of the uterus and the leiomyoma before embolization.

Late contrast enhanced scans are obtained at 14 institutions.

None of the institutions participating in the survey performs MR angiography as part of their routine protocol. Twelve sites perform MR angiography in selected cases, i.e. if embolization is a treatment option.

### Reporting

Standardised proforma reporting is only provided by 4/24 institutions. In the written report all institutions include the size, localisation, signal characteristics on T1W and T2W and differential diagnosis.

Consensus was reached that the presence of commonly coexisting adenomyosis needs to be clearly stated. In those cases, the thickness of the junctional zone should also be reported.

Except for 4 institutions, the size of the uterus is reported. Consensus was reached that the length of the cervix and corpus uteri, respectively, should be stated in the written report. Several institutions include all 3 dimensions of the uterine corpus.

The number of leiomyomas in the uterus is reported by all except 3 centres. Almost all (n=24/25) institutions report necrosis or degeneration as well as the perfusion pattern. In most cases (n=23/25), other findings in the abdomen will be reported. Lymph node enlargement or kidney obstruction as well as the distance of the fundus uteri to the umbilicus is reported for laparoscopic surgery.

Eleven institutions incorporate the FIGO classification of leiomyoma in their report.

### Imaging the pregnant patient

Almost half of the institutions (n=12/25) image patients with leiomyomas during pregnancy. Questions to answer are the relation to the placenta and the internal cervical os, assessment of acute abdominal pain and indeterminate sonography. During pregnancy, fast imaging sequences are used and Gadolinium is avoided.

## Discussion

The variety of responses to the questionnaire emphasises the need for this consensus statement. Practice clearly varies between institutions, sometimes on very basic issues. The authors intend that this article will help institutions understand the rationale behind currently considered best practice and this, in turn, will improve and unify practice.

### MRI Imaging

#### Patient preparation

Neither optimal scheduling according to the menstrual cycle nor a clinical questionnaire before MRI of uterine benign disease is covered in the recent literature. It is recommended that institutions do not schedule their patients according to their menstrual cycle, but obtain clinical information at the time of the scan (e.g., time of menstruation, clinical symptoms, hormonal medication, prior surgical procedures). This regimen obtained consensus among the ESUR Female Pelvic Imaging Working Group (Table 1).

**Table 1:** Patient Preparation

• Scheduling the exam according to the menstrual cycle is not necessary
**•** Clinical questions should be asked before the exam (e.g., time of menstruation, clinical symptoms, hormonal medication, prior surgical procedures)
**•** Fasting before the exam is recommended (3-6h)
**•** The use of antiperistaltic agents is recommended (20 mg butyl scopolamine im/iv or 1 mg of glucagon iv), unless their use is contraindicated due to patient medical background
**•** Patients should empty their bladder 1h prior to examination in order to achieve a moderately filled bladder

Despite lack of substantial evidence to support fasting for pelvic MRI examinations, there was agreement that patients should fast (3-6 hours), especially when intravenous (iv) gadolinium injection is considered. This is also supported by previous ESUR guidelines [3, 7, 8]. The rationale behind fasting is to reduce the risks from vomiting occurring as a reaction to the contrast medium injection as well as to reduce bowel motion artifacts.

There are no new data regarding the use of antispasmodic agents for the reduction of bowel artefacts. Its supporting evidence has also been extensively discussed in previous ESUR guidelines [3, 7–10]. The committee recommends the use of antiperistaltic agents for the improvement of image quality of pelvic MR (20 mg butyl-scopolamine im/iv or 1mg of glucagon iv), unless it is contraindicated.

There is consensus that a moderately filled bladder is optimal for pelvic MRI. The literature only sparsely addresses this issue. Previous ESUR guidelines recommend patients should empty their bladder 1h prior to examination to achieve a moderately filled bladder [8, 11–15].

#### MRI technical considerations (Table 2)

Although there are no studies comparing 3 Tesla versus 1.5 Tesla or pelvic array coils versus multi channel body coils in benign uterine lesions, all these options seem valuable.

**Table 2:** Proposed MR imaging protocol

MR BASIC PROTOCOL
**• Axial T1W of the pelvis** If high signal lesions are depicted, an axial FS T1W sequence should be performed
**• Sagittal and axial T2W of the pelvis or sagittal/oblique axial**
At least two T2W orthogonal oblique planes of the uterus, e.g. sagittal T2W sequence of the corpus of the uterus; Axial oblique T2W sequence of the corpus of the uterus
SPECIFIC CLINICAL SETTINGS
**• Work-up of infertility** **-** Add an oblique coronal T2W sequence along the long axis of the uterus to the basic protocol
**• Work-up of an adnexal mass of indeterminate origin** **-** Add an oblique coronal T2W sequence along the long axis of the uterus to the basic protocol **-** Add dynamic contrast-enhanced study to the basic protocol in the oblique coronal plane along the long axis of the uterus. Alternatively select a plane across the maximum point of contact between the mass and the uterus **-** If the mass shows to be ovarian follow the ESUR guidelines for characterization of indeterminate adnexal masses.
**• Work-up of a rapid growing uterine mass** **-** Add dynamic contrast-enhanced study to the basic protocol in the plane that best depicts the morphology of the lesion to be characterized **-** DWI study is optional
**• Work-up of an intermediate to high T2W leiomyoma** **-** Add dynamic contrast-enhanced study to the basic protocol in the plane that best depicts the morphology of the lesion to be characterized **-** DWI study is optional
**• Pre- and post-embolization assessment** **-** Add MR angiography or dynamic contrast-enhanced study to the basic protocol **-** Add DWI sequence to the basic protocol **-** MRA pre-embolization: evaluate the number, site of origin and size of uterine and ovarian arteries **-** MRA post-embolization is optional: useful in demonstrating eventual collateral supply to LM that where not fully devascularized

It is recommended that institutions routinely include at least one fast T2WI sequence of the upper abdomen. This strategy allows exclusion of complications in patients with large leiomyoma, e.g. signs of compression such as hydronephrosis, or to rule out lymphadenopathy or metastases in patient with suspected malignancy.

We recommend a basic MRI examination protocol with at least two T2WI orthogonal planes of the uterus, including a sagittal sequence of the uterine corpus. Doing the sagittal sequence first allows subsequent scan planes to be aligned to the axis of the uterus rather than just aligned to the body. An oblique sequence aligned along the long axis of the uterus is optional, but provides useful information for anatomic localization of leiomyomas, particularly in the work-up of infertility, as it best displays the relationship of the leiomyomas to the endometrial cavity. A 3D T2W sequence may be an alternative in selected cases, e.g. mapping in multiple myomas. In the work-up of an adnexal mass of indeterminate origin, a T2W plane selected across the maximum point of contact between the mass and the uterus is advised [10, 13, 16, 17] (Tables 2 & 3).

**Table 3:** MR imaging sequences and rationale

Sequences/technique	Diagnostic value	Literature
**Basic protocol**		7,10,12,13,16
Sag/Oblique axial T2W and T1W axial	Anatomy, characterisation and mapping of leiomyomas DDx adenomyosis Other findings	
Fast T2W upper abdomen	Large tumors, renal obstruction, metastases	
**Optional sequences**		
T1W FS	DDx of fatty from hemorrhagic lesions of the uterus (e.g lipoleiomyomas, leiomyomas with haemorrhagic degeneration or haematometra) and the ovaries (teratoma, endometriomas)	10,13,16,17
Oblique coronal T2W	Relationship to uterine cavity, DDx of uterine (claw sign) and ovarian origin	3,10
Gadolinium T1W (optimally DCE)	Characterisation of leiomyomas DDx from leiomyosarcomas and adnexal masses (bridging feeding vessels); pre- and post embolisation therapy	12,13, 17-23,
DWI	Characterisation in atypical leiomyomas; treatment in leiomyoma embolisation	11, 28-37

Besides the T2W sequences, the basic protocol should include an axial T1W sequence in order to assess other pelvic pathologies and to depict high-signal intensity lesions. In this scenario, a fat-supressed T1W sequence in the same plane allows characterization of haemorrhagic from fatty uterine pathologies (lipoleiomyomas vs leiomyomas with haemorrhagic degeneration; haematometra; endometriosis and adenomyosis), adnexal masses (teratomas or endometriomas) or other pelvic fat or blood-containing masses [10, 13, 16, 17] (Table 3).

Major indications for iv gadolinium include: characterization of a rapidly growing leiomyoma; characterization of a leiomyoma with areas of high signal intensity on T2W images; differentiation from an adnexal mass; pre-embolization assessment including vascularization and viability of leiomyomas; post-embolization assessment to define treatment response in persistence of clinical symptoms or post-embolization complications [12, 17–23] (Table 3).

Gadolinium is optimally administered in a dynamic acquisition in the plane that best depicts the morphology of the lesion to be characterized, as determined based on the T2W sequence.

Especially for radiologic interpretation of therapeutic response, image subtraction can be helpful in distinguishing enhancing areas from T1W hyperintense material such as haemorrhage or debris.

**Fig. 1:**
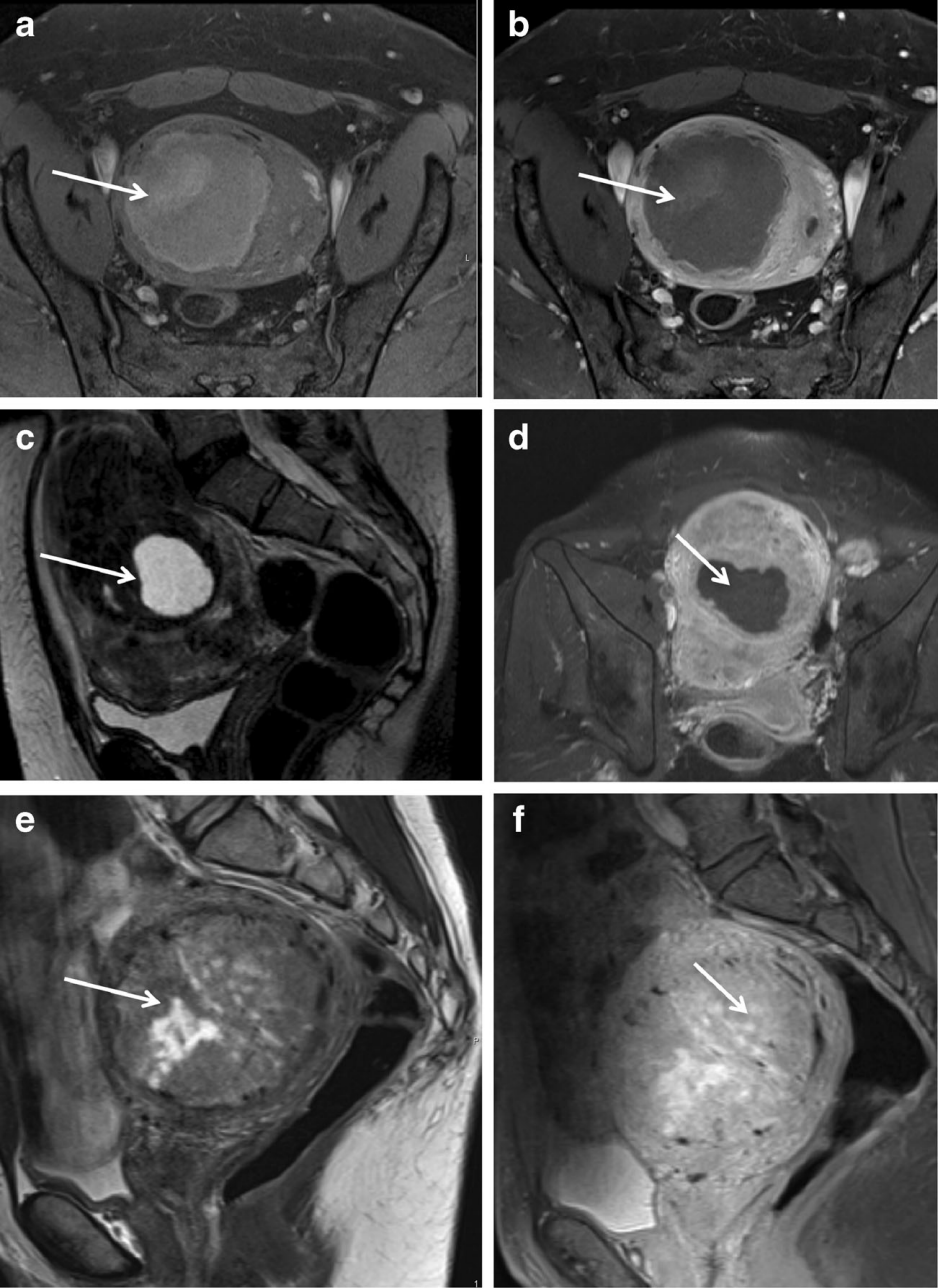
Different aspects of leiomyoma degeneration: **Hemorrhagic degeneration **after UFE. Axial T1-weighted fat saturated images, before (**a**) and after gadolinium administration (**b**) show a leiomyoma with increased signal on T1 and lack of enhancement, consistent with hemorrhagic degeneration. **Cystic degeneration**. Sagittal T2-weighted (**c**) and axial T1-weighted fat saturated contrast enhanced (**d**) images show a leiomyoma presenting a peripheral area with high T2 intensity signal and no enhancement, consistent with cystic degeneration (arrows). **Myxoid degeneration**. Sagittal T2-weighted (**e**) and T1-weighted fat saturated contrast enhanced (**f**) images show a leiomyoma presenting areas with high T2 intensity signal and enhancement after gadolinium administration (in contrast with the cystic degeneration), suggesting myxoid degeneration (arrows)

Different types of benign degenerating leiomyomas show T2W hyperintense areas and can rapidly grow (cystic, myxoid, hydropic and cellular degeneration) [23–25]. In these settings, an intravenous contrast medium is imperative to identify solid components of leiomyosarcomas, which may also show rapid growth and T2W hyperintensity due to necrosis (Fig. 1). Unfortunately, differentiation between benign degenerating cellular leiomyomas and leiomyosarcomas is challenging due to considerable overlap [12, 13, 23] (Table 4). Lakhman et al recently reported four discriminitative features: nodular borders, haemorrhage, T2W dark areas and central unenhanced areas. Combination of >3 MR features allowed specificity of >95% [26].

**Table 4:** Differentiation between leiomyoma and leiomyosarcoma

	Leiomyoma	Leiomyosarcoma
**Age**	Premenopausal	Peri/postmenopausal
**Borders**	Well delineated	Often nodular*
**DWI**	Variable	Restricted diffusion, low ADC
**Invasiveness**	No	Adjacent tissues
**Number**	Commonly multiple	Solitary
**Size**	Variable	Large (>10cm)
**T2WI**	Mostly low, high in degeneration, whorled pattern	Inhomogenous with areas of hemorrhage*, intralesional vessels; T2 dark areas*
**Vascularity**	Variable; often parallels myometrium, cellular types with avid enhancement	Hypervascularization; peripheral early enhancement, central necrosis*

* ≥3 of *features: 95% specificity in predicting leiomyosarcoma according to Lakhman and al (features are highlighted in bold) (26)

In a T2W hypointense solid mass of indeterminate origin, studies have shown that ovarian fibromas enhance late and less compared to the myometrium, whereas typical subserosal leiomyomas tend to have a similar enhancement pattern [18, 19].

**Fig. 2:**
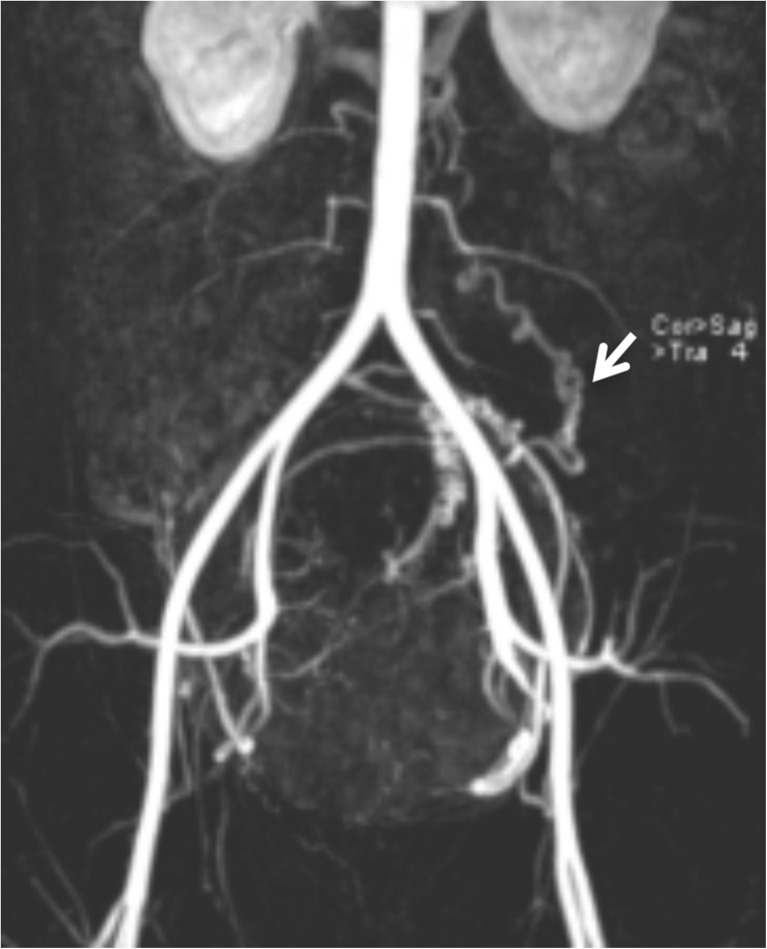
Ovarian artery parasitation. 3D reconstructed MRA image depicts an enlarged left ovarian artery (arrow) extending from the aorta to the pelvic midline, where a bulky leiomyoma is located, apparently contributing to the leiomyoma supply. Note the typical corkscrew appearance of the ovarian artery. The right ovarian artery is not seen as the normal caliber ovarian arteries are too small to be depicted by this imaging modality

3D-reconstructed contrast-enhanced MR angiography, has proven to be valuable in determining details of the pelvic vasculature and the optimal image intensifier C-arm obliquity before embolization, thus reducing the fluoroscopy time, the radiation dose and the contrast medium volume (Fig. 2) [20–22]. Currently, MR angiography is not part of the routine protocol. Twelve of the participating institutions perform MR angiography in selected cases.

**Fig. 3:**
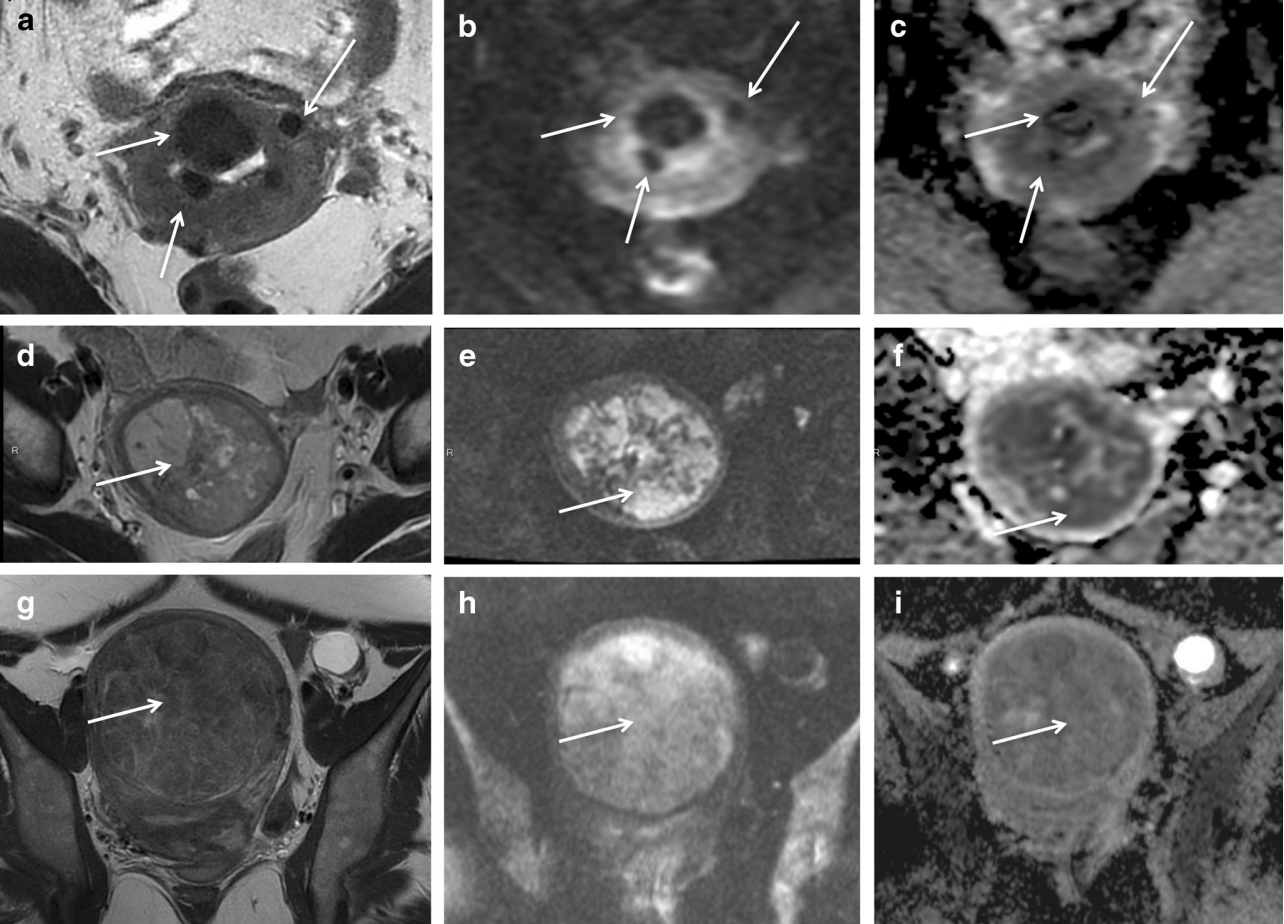
Leiomyomas and DWI. Oblique-axial T2-weighted (**a**) and b=800 diffusion-weighted (**b**) MR images and apparent diffusion coefficient map (**c**) of pelvis show multiple uterine leiomyomas (white arrows) that are hypointense in all three sequences, the so-called T2 blackout effect. (**d**) Axial T2-weighted MR image shows a heterogeneous hyperintense lesion (white arrow). (**e**) Axial diffusion-weighted MR image at b=800 shows large areas of increased signal intensity (white arrow) in mass. (**f**) Apparent diffusion coefficient map shows restriction (white arrow). The measured ADC is 0.7. Histopathologic examination confirmed the diagnosis of leiomyosarcoma. (**g**) Oblique axial T2-weighted MR image shows a well-defined lesion (white arrow) of intermediate to high signal intensity. (**h**) and (**i**), Oblique-axial diffusion-weighted image (b=800) shows high signal intensity (white arrow) and restriction on the corresponding ADC map (white arrow), with a measured ADC of 1.25. Histopathologic result was cellular leiomyoma

Based on the current literature DWI is not mandatory for assessing leiomyomas in a clinical routine setting. However it might have a potential for characterization of T2W hyperintense leiomyomas or in rapid growth (Fig. 3). Both leiomyosarcomas and benign cellular leiomyomas display restricted diffusion. Thus, restricted diffusion in a leiomyoma is a pitfall of which radiologists should be aware [11, 27–29]. Two publications found significantly lower mean ADCs in leiomyosarcomas than in degenerated leiomyomas (Fig. 3) [30, 31]. As reported previously by our working group, for gynaecological imaging, the optimal b value is usually 800–1000 s/mm^2^, but may be increased up to 1200 or 1400 s/mm^2^ [10].

DWI has also been shown to be useful in pre- and post-embolization evaluation of uterine leiomyomas. Persistent decrease of the ADC values of the dominant leiomyoma 6 months following embolization is reported, which also correlates with its volume reduction [32–37]. Therefore, a high pre-embolization ADC value seems related with a greater volume reduction and to be useful in predicting the response of a dominant leiomyoma to embolization [33–35].

#### Reporting

Although standardized pro forma reporting was used in only a few institutions, there was consensus that this will become an issue in the future and that the written report has to include mandatory findings (Table 5).

**Table 5:**
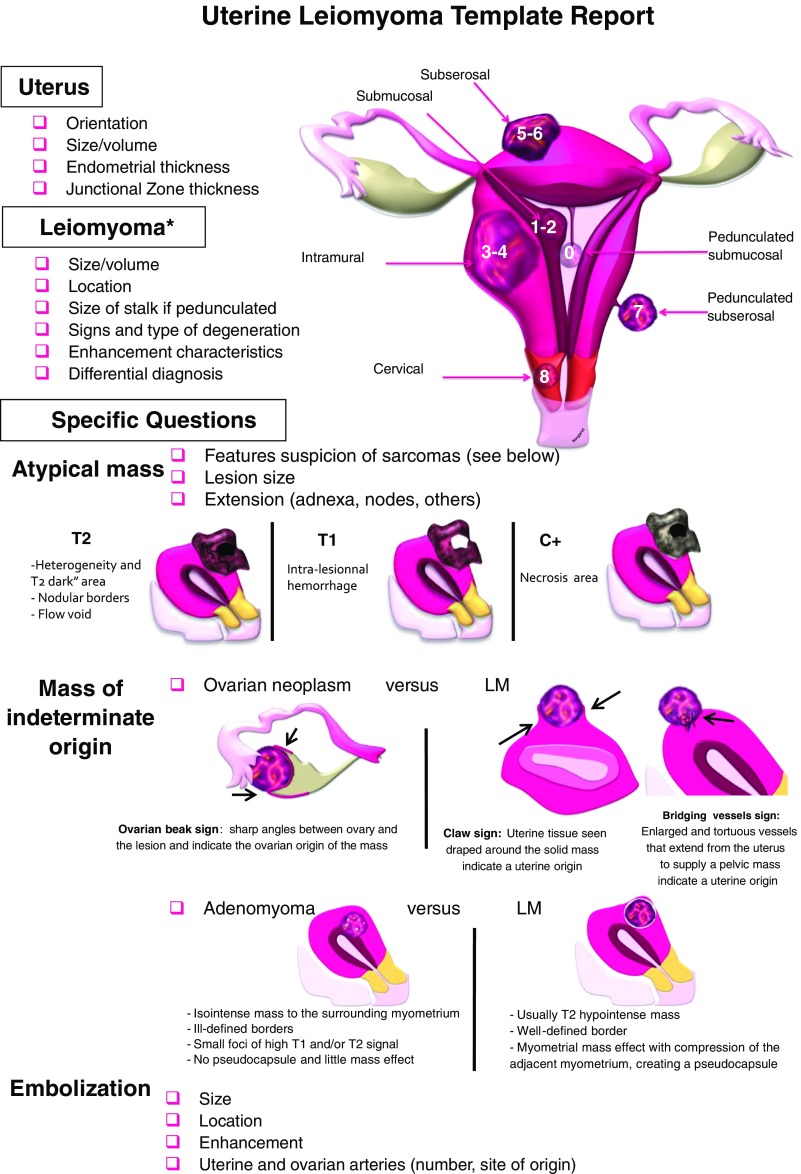
Proposed leiomyoma reporting template

A standard template report dedicated to uterine leiomyomas MR imaging is proposed by the Female Imaging Working Group, listing the relevant items to mention in the report. The main differential diagnosis and the diagnostic clues are illustrated

(LM – leiomyomas)

In case of multiplicity, evaluation should be performed on a per-patient basis: only atypical ones should be described. If there is no atypical leiomyoma, the largest leiomyoma should be measured

The FIGO classification is reported on the drawing (FIGO 0: pedunculated intracavitary; FIGO 1: <50 % intramural; FIGO 2: ≧ 50% intramural; FIGO 3: contacts endometrium, 100 % intramural; FIGO 4: intramural; FIGO 5: subserosal ≧ 50% intramural; FIGO 6: subserosal <50 % intramural; FIGO 7: subserosal pedunculated; FIGO 8: other (specify eg, cervical, parasitic)

Differentiation between leiomyoma and ovarian fibroma, adenomyoma and leiomyosarcoma are also highlighted

**Fig. 4:**
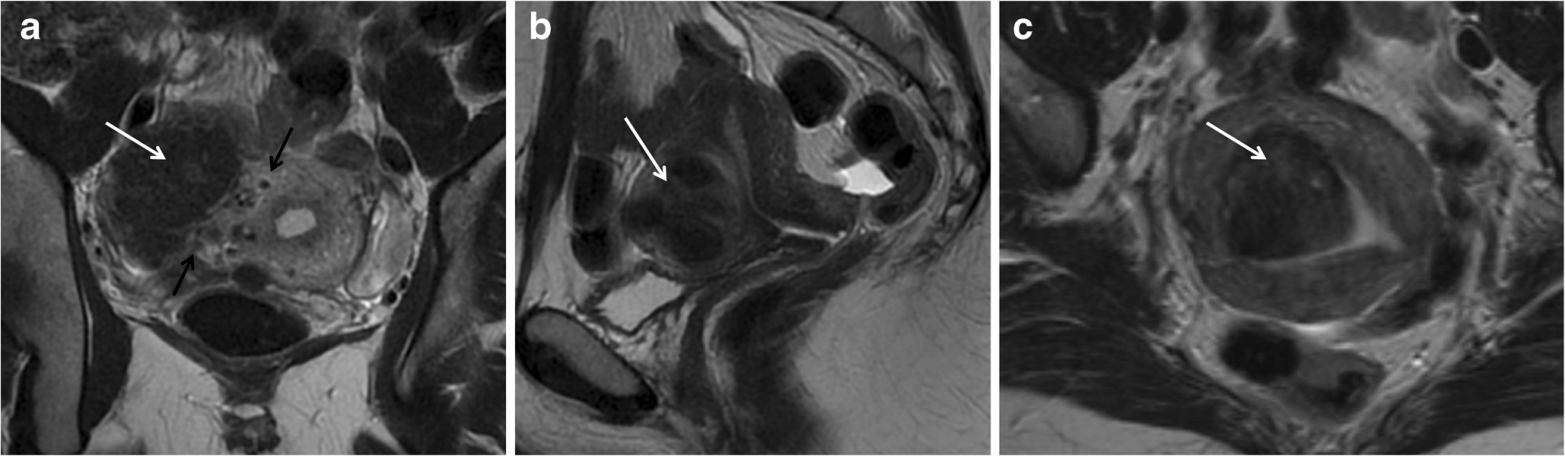
Leiomyoma FIGO classification according to LM location: (**a**) Axial oblique T2-weighted image showing a subserosal leiomyoma being less than 50% intramural (MRI FIGO 6) (white arrow). Note the bridging vessels feeding the lesion (black arrow). (**b**) Sagittal T2-weighted image showing an intramural leiomyoma (MRI FIGO 4). (**c**) Axial oblique T2-weighted image shows a submucosal leiomyoma being less than 50% intramural (MRI FIGO 1)

**Fig. 5:**
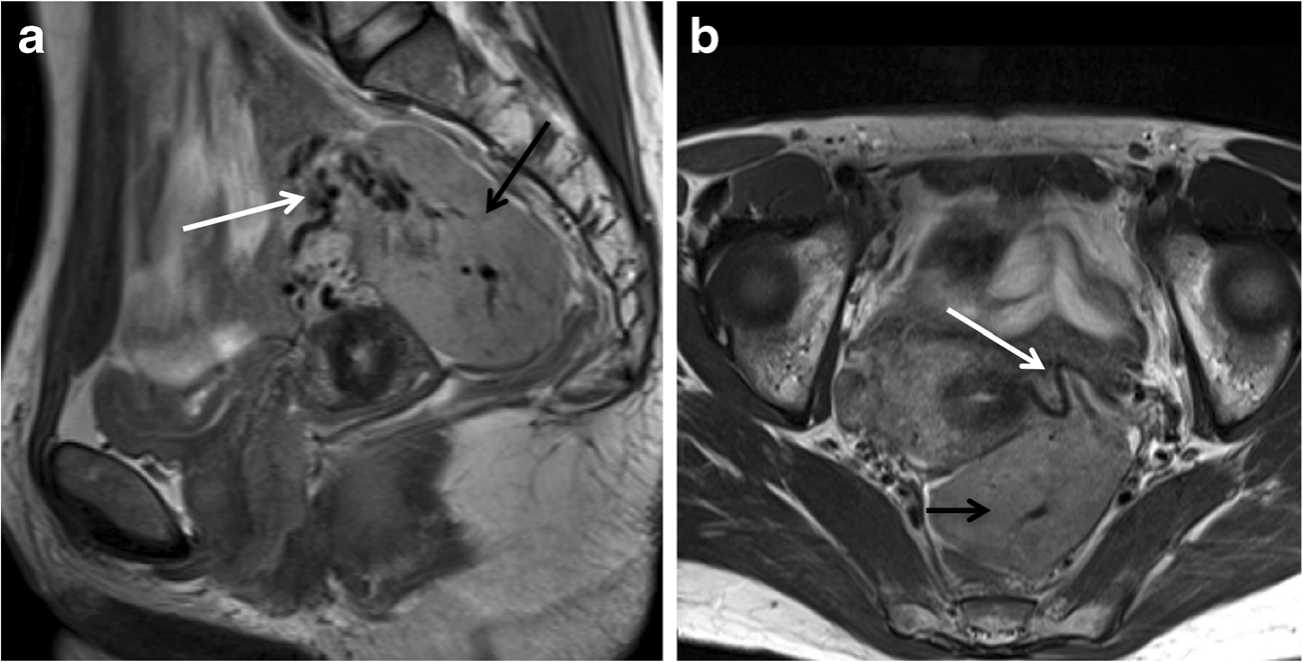
Sagittal and axial T2-weighted images showing a pedunculated subserosal leiomyoma (MRI FIGO 7) within the pouch of Douglas (black arrow). The bridging vessels sign confirms the uterine origin of the mass (white arrow)

Leiomyomas are typically multiple and are classified according to their location as submucosal, intramural and subserosal (Figs. 4 and 5). To standardize the nomenclature in patients with abnormal bleeding, regardless of their number and size, they can be further subdivided into 8 categories according to the FIGO classification (PALM-COEIN) (Table 5) [38, 39].

Size and MR features, e.g. signs of necrosis or degeneration as well as enhancement patterns, must be described (Table 5) [1].

Unusual variants, such as diffuse leiomyomatosis characterized by symmetrical enlargement of the uterus due to innumerable small ill-defined leiomyomas, lipoleiomyomas, and parasitic leiomyomas should be mentioned in the report [1, 2].

**Fig. 6:**
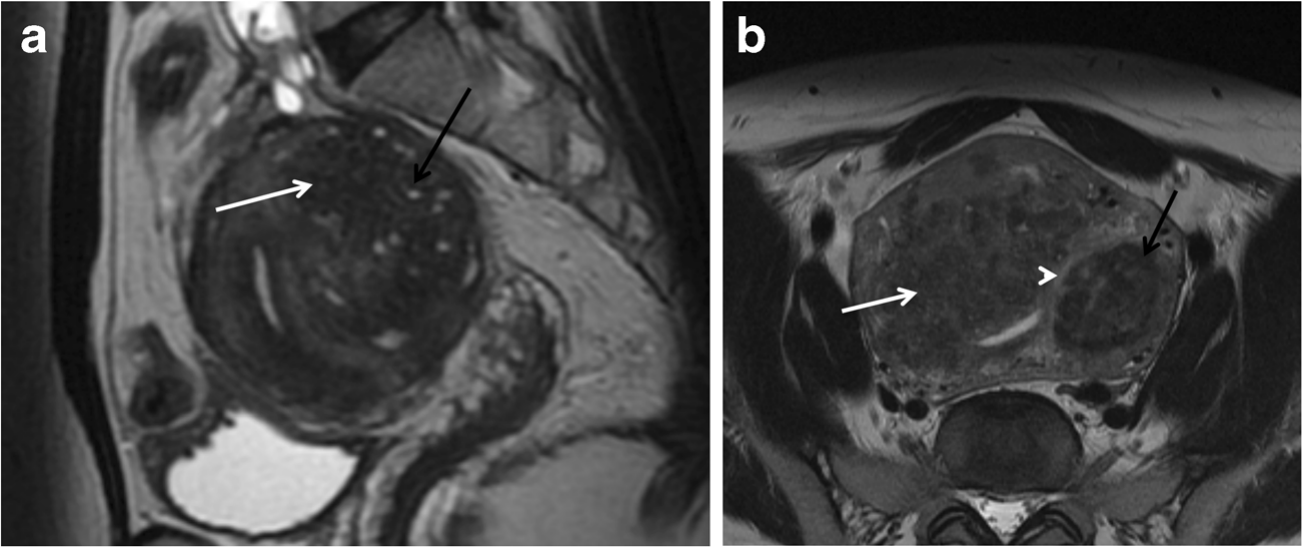
Differences between leiomyomas and adenomyoma/adenomyosis. (**a**) Sagittal T2W image shows a poorly defined border, oval-shaped, low-signal mass (white arrow) with hyperintense T2 foci embedded in the lesion (black arrow), consistent with an adenomyoma. (**b**) Axial oblique T2W image demonstrates an ill-defined thickening of the junctional zone with hyperintense T2 foci, consistent with adenomyosis (white arrow). Nearby there is a T2 hypointense lesion, with a well-defined margin in keeping with an intramural leiomyoma (black arrow). Notice the thin T2 hyperintense rim surrounding the lesion, indicating a pseudocapsule of edema secondary to some degree of venous or lymphatic obstruction, typical for leiomyomas (arrowhead). Coexistence of adenomyosis and leiomyomas is not rare

Inclusion of any differential diagnosis is mandatory in every report. These include myometrial contractions, adenomyosis (Fig. 6), and, most importantly, uterine malignancy or adnexal fibromas.

Depending on the planned procedure other imaging information may be required (Table 5)*.*

#### MRI for treatment planning and therapy monitoring

While (as corroborated by our survey) currently laparoscopic or open surgery is still the mainstay of operative intervention, minimal invasive interventions, such as Uterine Leiomyoma/Fibroid Embolization (UFE) and Magnetic Resonance guided Focused Ultrasound (MRgFUS) are emerging techniques that are likely to gain increasing acceptance. In 14 of the participating institutions, embolization is offered to patients, while only one institution had experience with MRgFUS.

Although not mandatory for pre-UFE evaluation using high-quality ultrasonography, MRI may change diagnosis and treatment plan in about 20% of patients with symptomatic leiomyomas [4–6]. Patients with leiomyomas and coexisting adenomyosis are potential candidates for uterine artery embolization. The coexistence of adenomyosis should be reported, as it may require changes in the embolization protocol and might have a negative impact on long term results [4, 40–42]. Further, the size, number and location of leiomyomas are important for choosing treatment options for the individual patient. Though not contraindicated for UFE, it is valuable to visualize pedunculated leiomyomas for pre-treatment planning [4, 43, 44]. Cervical leiomyomas may not have a beneficial outcome after UFE [45]. Finally, it is crucial for the outcome of UFE that leiomyomas are vascularized, since a treatment response cannot be expected in a degenerated leiomyoma. MR-angiography (MRA) identifies major arteries supplying the leiomyomas. Usually the uterine arteries are the dominant suppliers, but blood supply by ovarian arteries may be a concern [4]. In these cases, the ovarian arteries are visualized as enlarged winding vessels on the MR angiogram (Fig. 2), while they are not clearly visualized under normal circumstances [46].

After UFE, leiomyomas usually undergo degeneration, although some observations contradict this [33, 47, 48]. If enhancement persists, repeat embolization of collateral blood supply or other treatment options may be considered [34, 45].

Post UFE problems may be identified by MRI. Persistent vaginal discharge may indicate a necrotic remnant or, rarely, expulsion and transvaginal sloughing of a necrotic leiomyoma may occur [4, 49].

MRgFUS induces focal heating and coagulative necrosis within a leiomyoma under real-time MRI monitoring using thermal maps [50, 51]. Ideally, leiomyomas should be limited in number and size (<10cm in largest diameter), show homogenously low-signal on T2W and no calcifications. Pre-treatment MRI in prone position allows targeting of a safe pathway during MRgFUS. Abdominal scars or pedunculated leiomyomas are relative contraindications, and intestine along the acoustic pathway is an absolute contraindication. Post treatment predictor of success is the non-perfused volume of the leiomyoma. Treatment typically achieves around 50–55% non-perfused volume, based on post contrast imaging after the completion of treatment [52].

## Conclusion

MRI offers an outstanding and comprehensive map of the size, site and distribution of leiomyomas. A standardised imaging protocol and method of reporting ensures that the salient features are recognised and recorded. All radiologists involved in the assessment of leiomyomas need to know the varied imaging features found on MR and their significance. It should go without saying that a good working relationship between the radiologist and the gynaecologist is essential in maintaining the quality of an imaging service. Such a relationship means images can be discussed and clinical feedback given on outcomes.

Our questionnaire has shown a reasonable homogeneity of practice across institutions. It has allowed the ESUR working party to draw together a consensus of best imaging practice and formulate these guidelines.

## Electronic supplementary material

ESM 1 (DOC 8.58 mb)
