# Supplementary material for: European Society of Urogenital Radiology (ESUR) Guidelines: MR Imaging of Leiomyomas
Source: Eur Radiol. 2018 Feb 28;28(8):3125–37. doi: 10.1007/s00330-017-5157-5 (PMC6028852; doi:10.1007/s00330-017-5157-5)
Supplement: Supplementary file 1 — (DOC 8.58 mb) [file 330_2017_5157_MOESM1_ESM.doc]

**Uterine Leiomyoma Template Report**

**1A 1B**

**
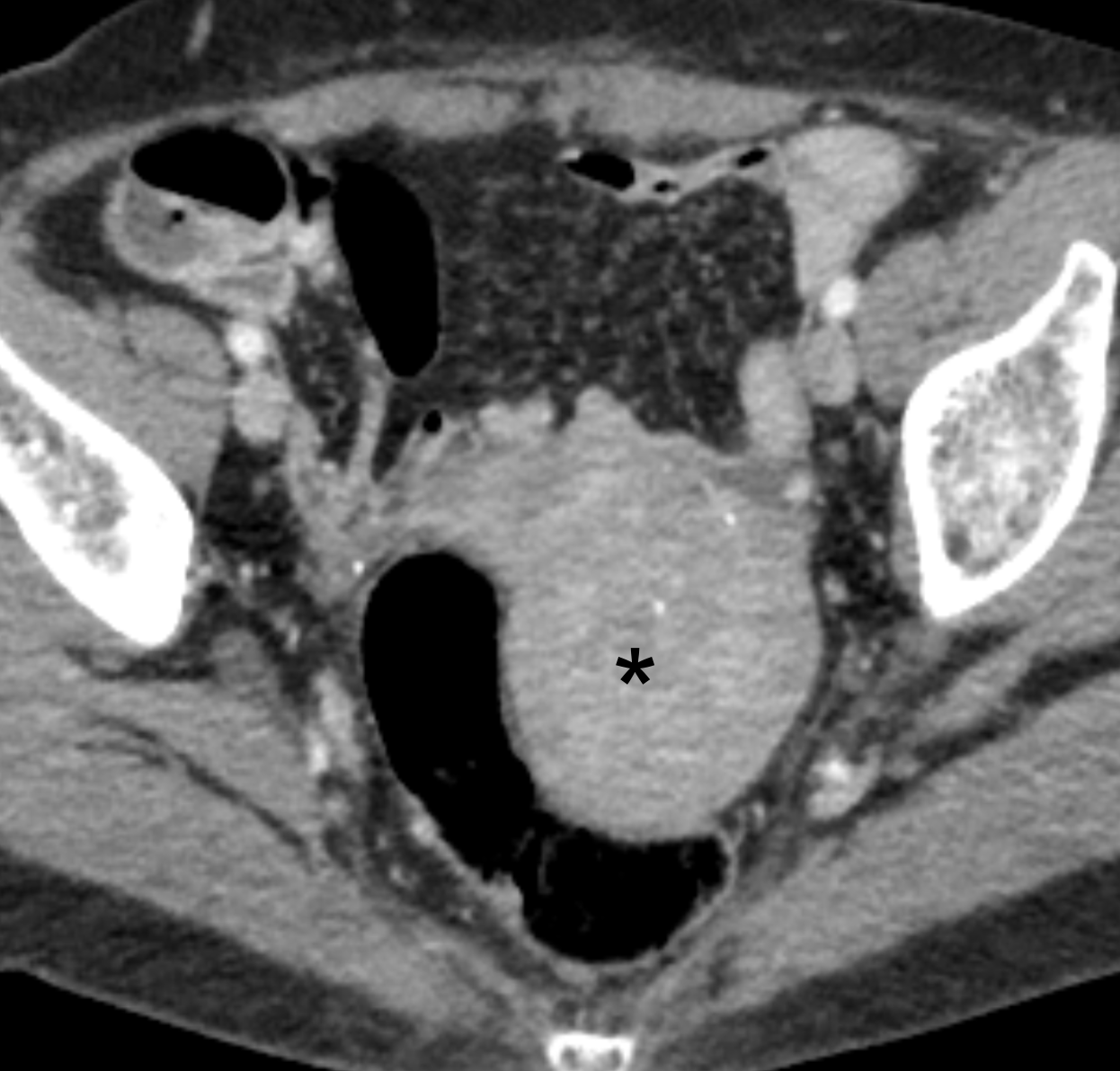

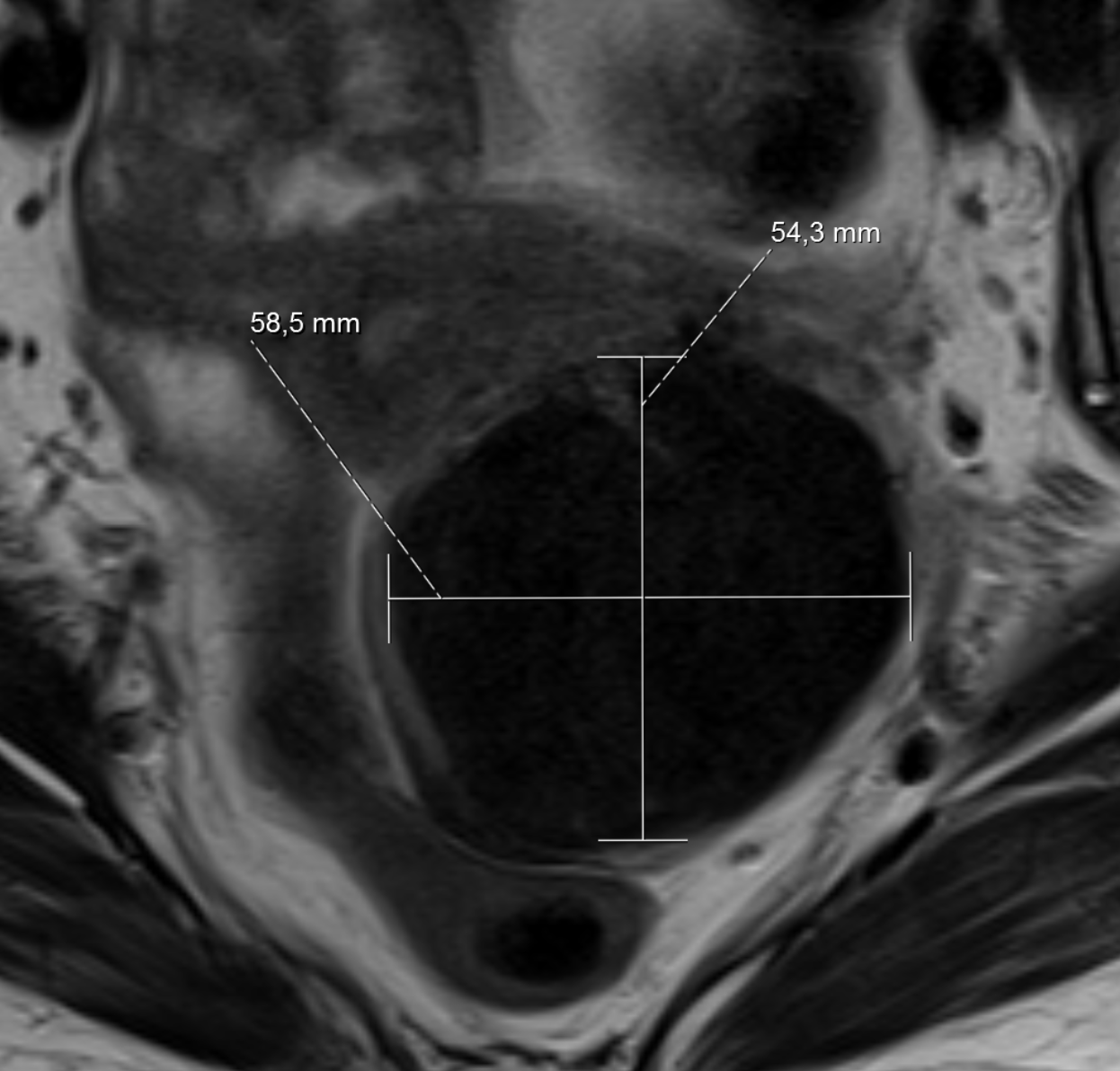
**

**1C 1D**

**
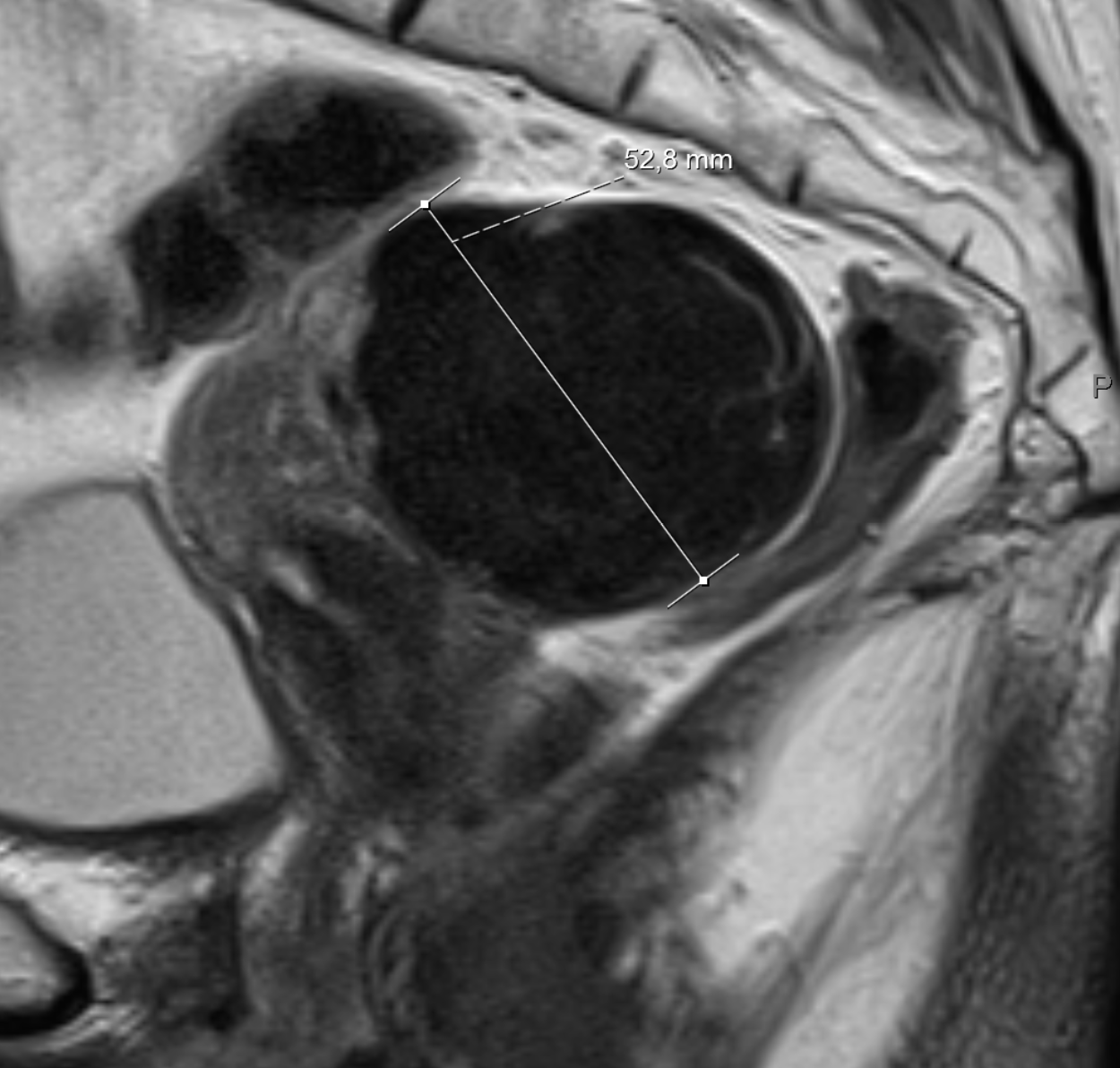

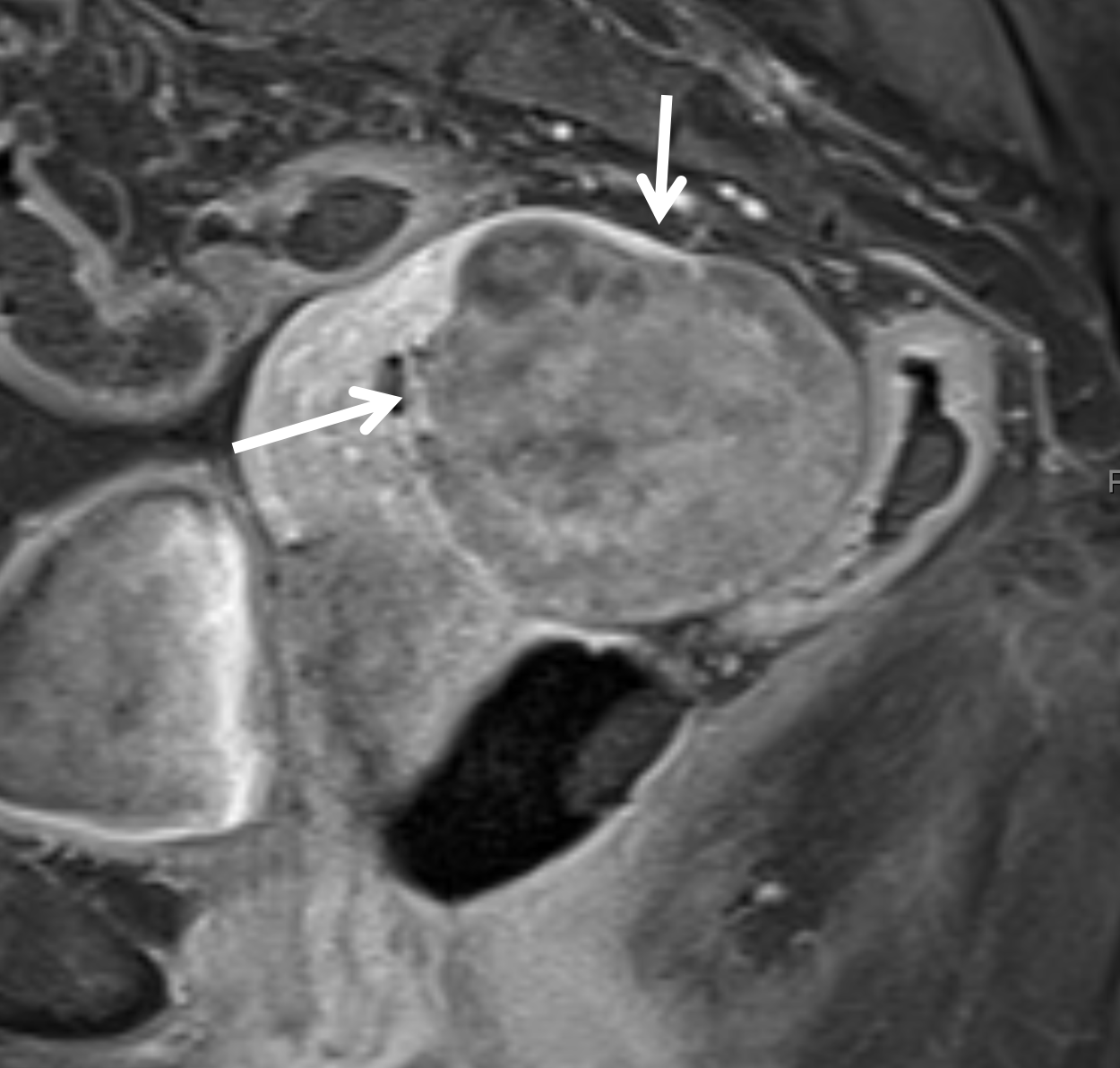
**

**Supplementary Files – 1**

**The claw sign.** A pelvic solid mass was incidentally found in a CT examination of a 65-year-old woman and a pelvic MRI followed for better characterisation. Axial CT image (A) shows a solid, round pelvic mass (asterisk) of indeterminate origin. Axial and sagittal T2W images (B, C) show a T2 dark solid mass within the pouch of Douglas in close relation to and behind the uterus. A claw of normal myometrium grasping this mass is clearly demonstrated in the fat-suppressed contrast-enhanced T1W image (arrows in D) confirming uterine origin. The imaging findings are consistent with a subserous leiomyoma.

The volume of the leiomyoma was easily estimated as 88 cc using the simplified ellipsoid formula 0.52 × length × width x depth.

**Template:**

**Uterus:**

**Orientation:** Anteverted-retroflexed

**Size/Volume:** 5.7 x 2.6 x 4.6 cm/35cc (not including the LM)

**Endometrial thickness:** 4mm

**Junctional zone thickness:** JZ is not clearly seen

**Leiomyoma**

**Size/Volume**: 5.9 x 5.4 x 5.3 cm/ 88cc

**Location:** subserosal – type 6

**Size of stalk if pedunculated:** NA

**Signs and type of degeneration:** NA

**Enhancement characteristics:** Homogeneous and less than the myometrium.

**Differential diagnosis:** Mass of indeterminate origin. The presence of the claw sign indicates uterine origin.

**Features suspicious of sarcomas:**

**- Heterogeneity and T2 dark area:** No

**- Flow voids:** No

**- Intra-lesional hemorrhage:** No

**- Necrosis area:** No

**2A 2B**

**
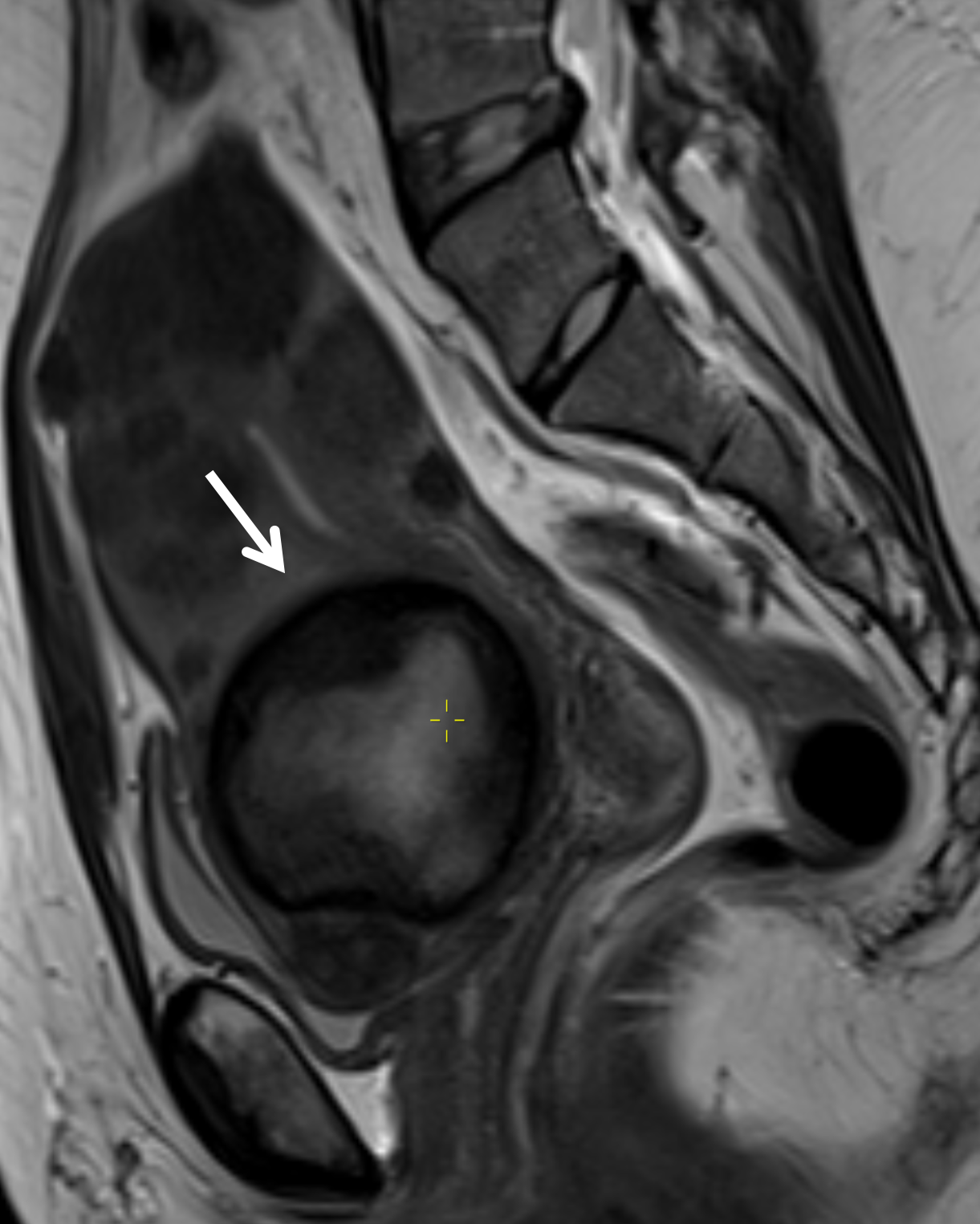

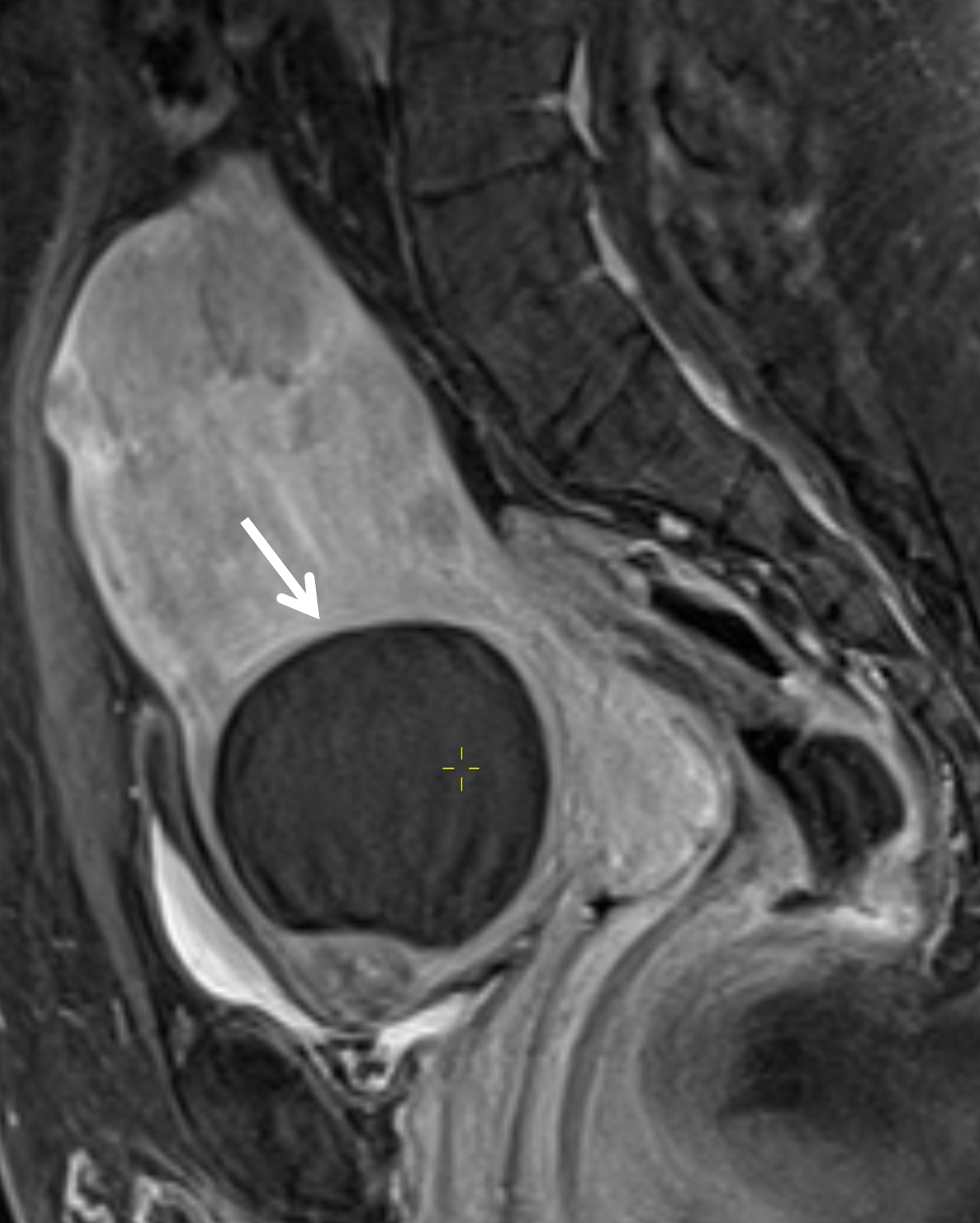
**

**Supplementary Files-2**

**Non-viable leiomyoma** in a 43-year-old woman presenting for preembolization assessment. Sagittal T2W image (A) depicts an enlarged uterus showing several leiomyomas. There is a dominant one (arrows) in the anterior wall of the uterine body, showing heterogeneous structure with internal areas of high T2 signal. After gadolinium administration (B) the dominant leiomyoma reveals complete lack of enhancement, consistent with self-infarction**,** while the smaller leiomyomas enhance homogeneously. Leiomyomas that have already infarcted are unlikely to show volume reduction with uterine artery embolization and, therefore, improvement of symptoms is less likely **with this treatment modality.**

**Template:**

**Uterus:**

**Orientation:** Anteverted-anteflexed

**Size/Volume:**  12.9 x 7.6 x 7.3 cm/475cc

**Endometrial thickness:** 3mm

**Junctional zone thickness: 6mm**

**Leiomyomas**

**Size/Volume**: 4.1 x 3.6 x 3.8 cm/29cc (dominant LM); poly-myomatous uterus

**Location:** Intramural – type 3 (dominant LM); intramural – type 4 and subserosal – type 5.

**Size of stalk if pedunculated:** NA

**Signs and type of degeneration:** NA

**Enhancement characteristics:** Lack of enhancement of the dominant LM; homogeneous enhancement of the other LMs.

**Embolization:**

Although the dominant LM was auto-infarcted, there were several viable fibroids. The clinical decision was to perform UAE for fibroid treatment.

**Uterine arteries:** Co-dominant

**Ovarian arteries:** Not enlarged

**3A 3B**

**
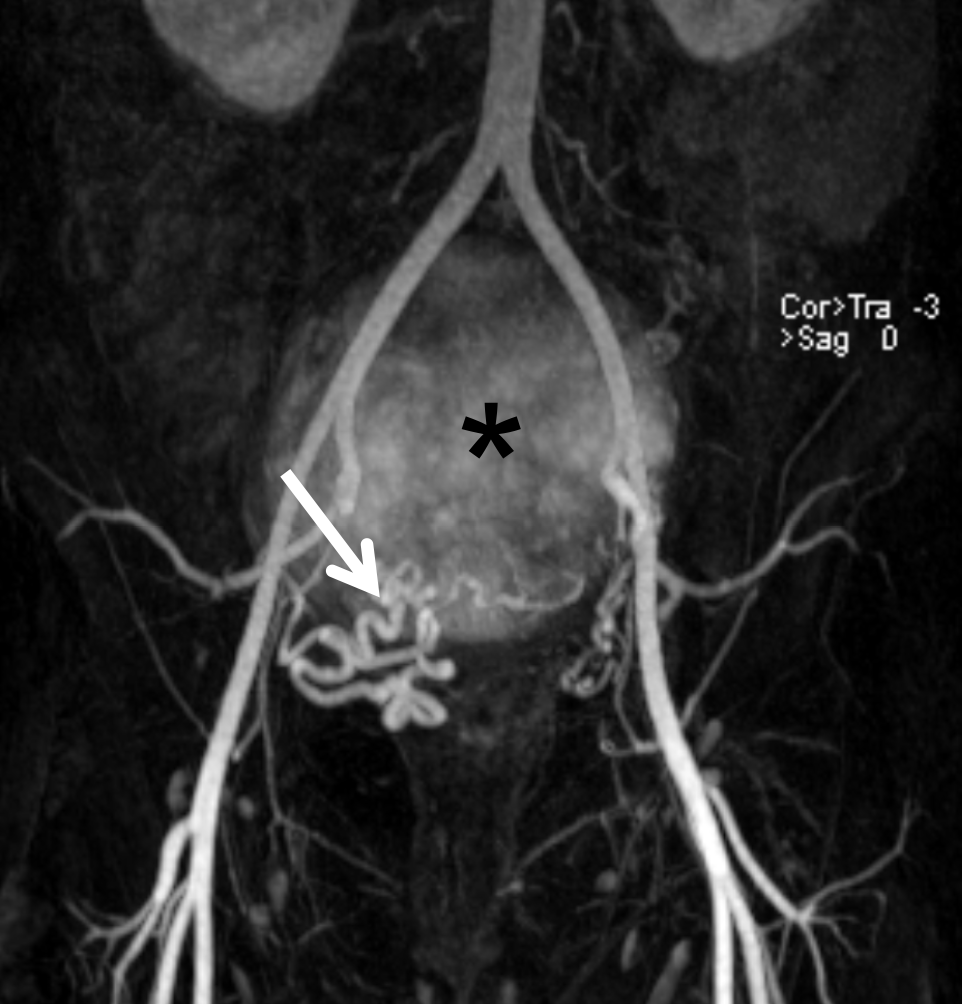

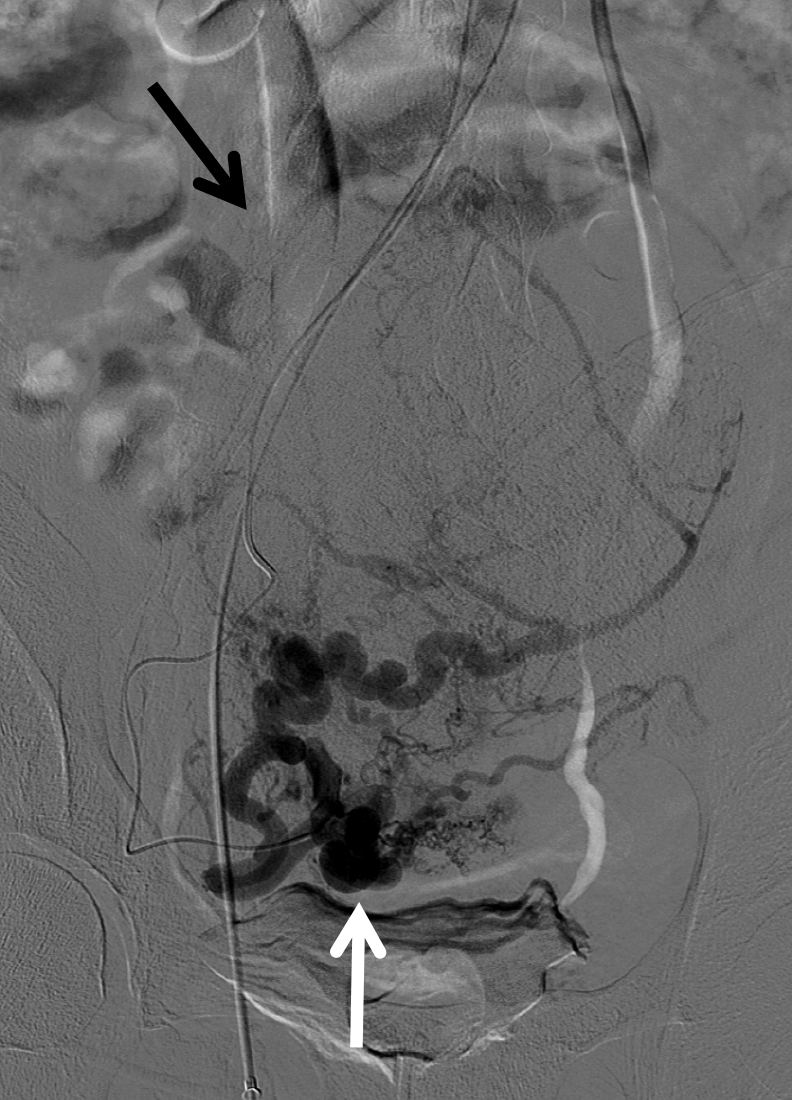
**

**Supplementary Files-3**

**MRA in preembolization assessment**.  3D reconstructed MRA image (A) shows the presence of both uterine arteries, with a dominant and prominent right uterine artery (arrow) and a normal caliber left uterine artery. No enlarged ovarian arteries are demonstrated.  A leiomyoma located in the midline is seen (asterisk). DSA (Digital Subtraction Angiography) image (B) obtained with right uterine artery selection has good correlation with the pre-procedural MRA image, showing the enlarged and tortuous right uterine artery (arrow) supplying the leiomyoma. DSA image depicts right hydronephrosis (black arrow), likely due to the ureteral compression by the bulky leiomyoma.

**4A 4B**

**
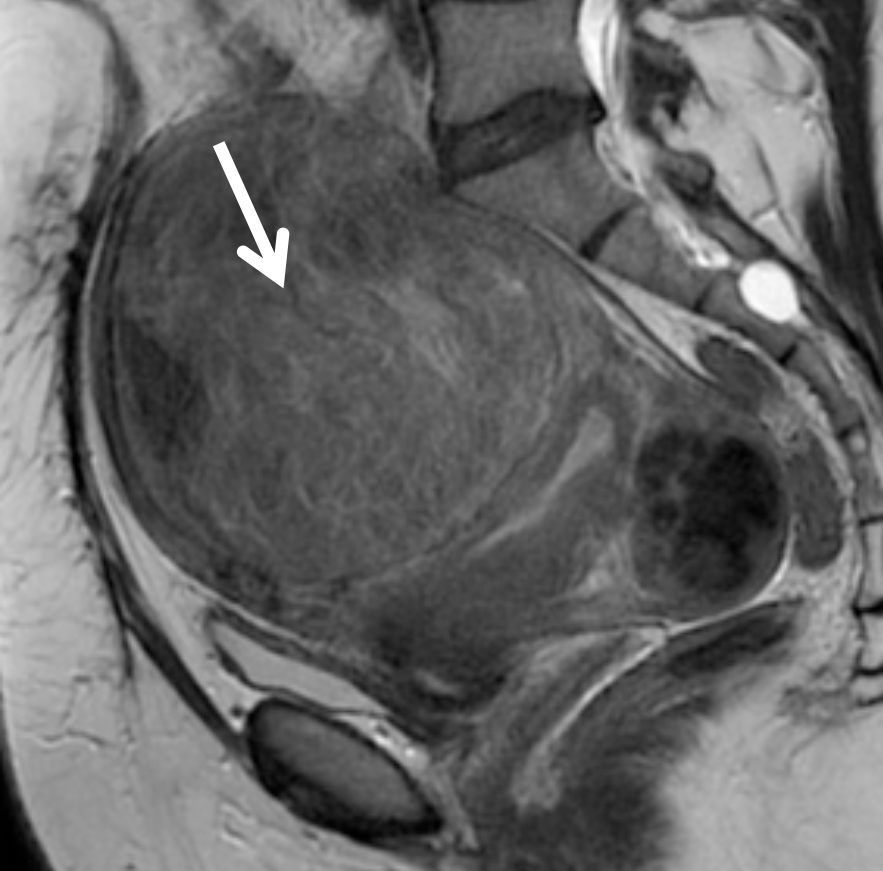

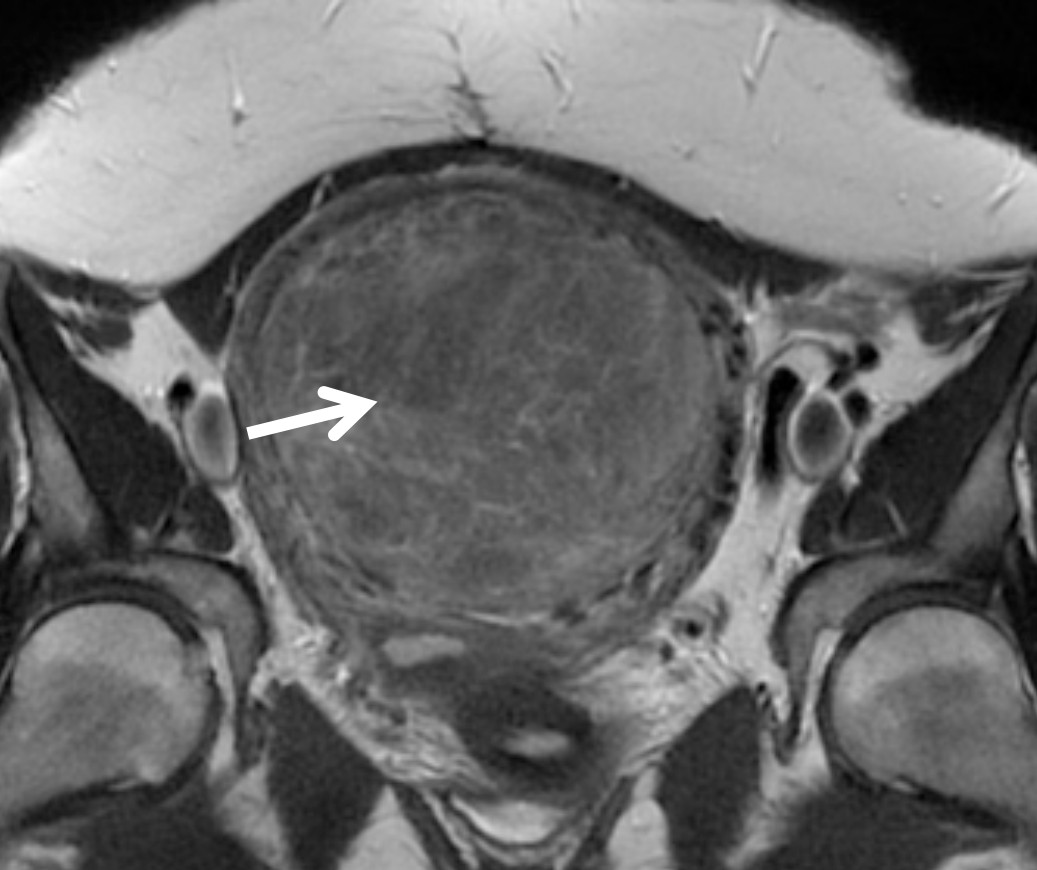
**

**4C 4D**

**
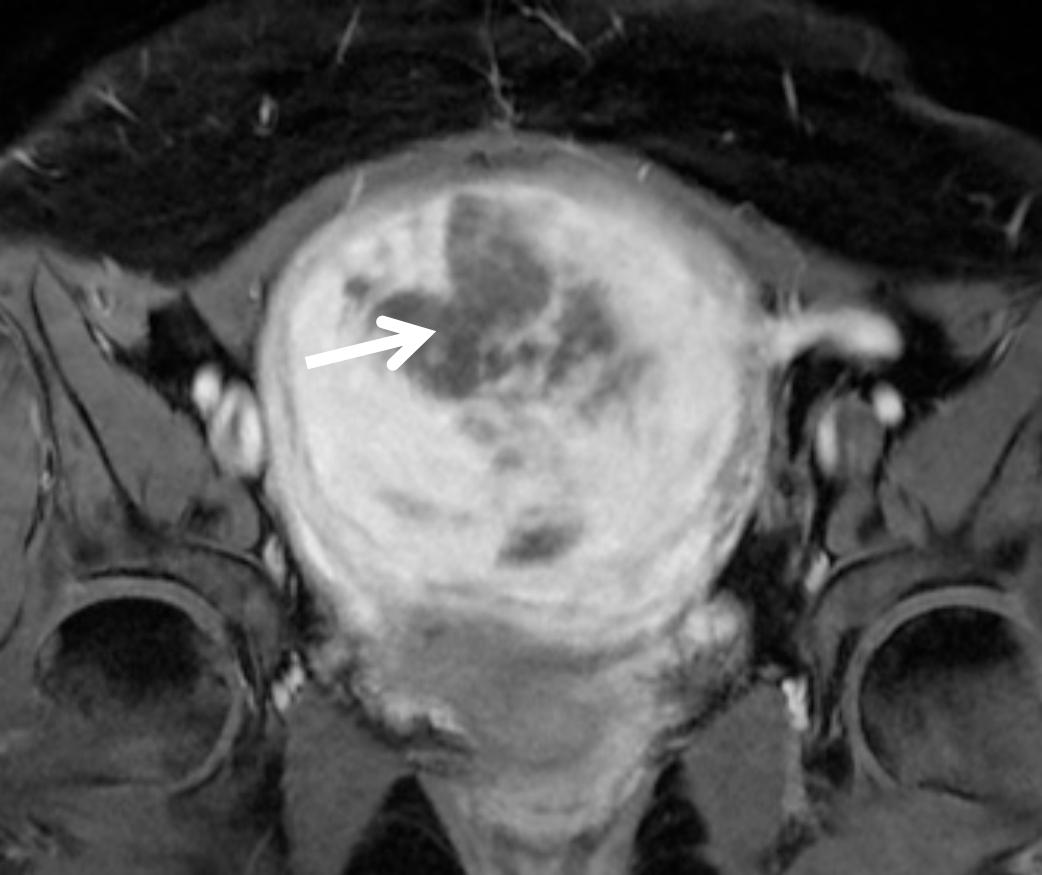

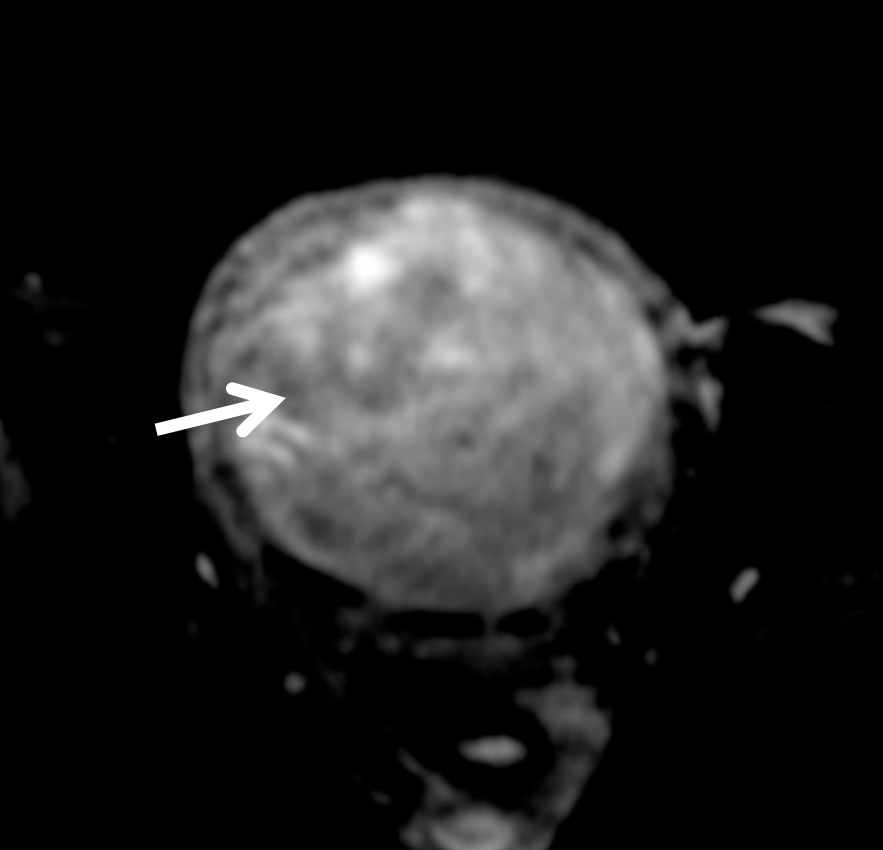
**

**4E**

**
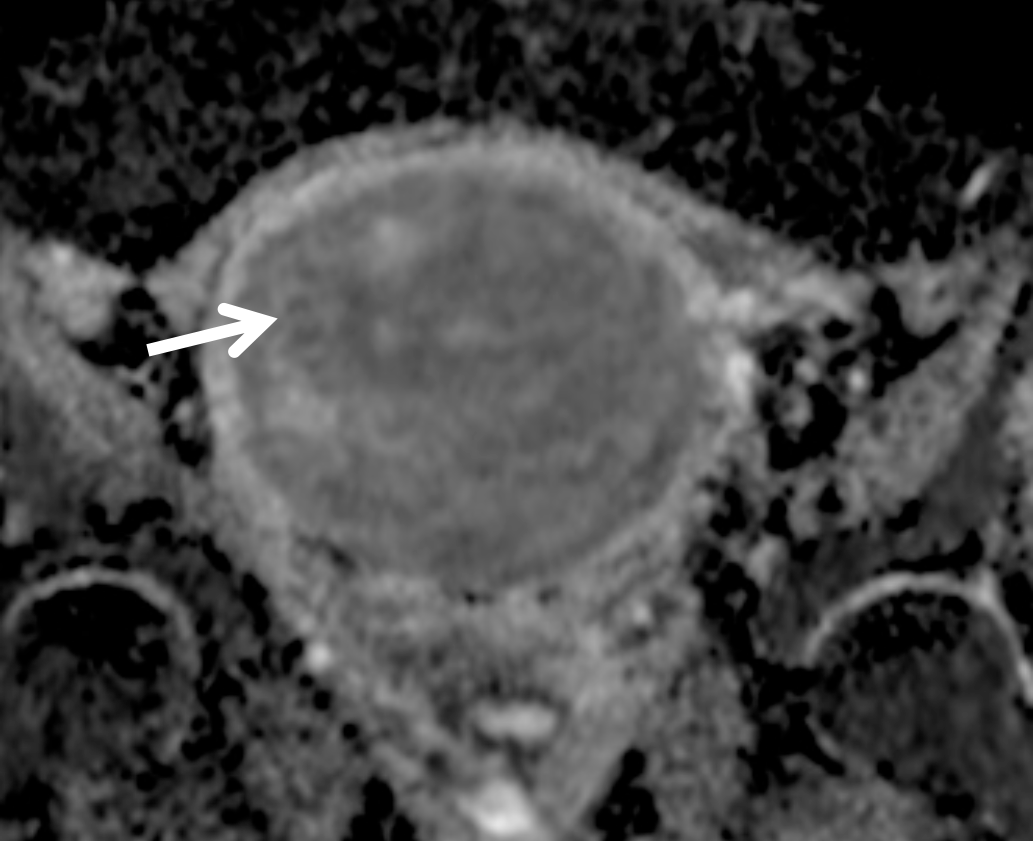
**

**Supplementary Files-4**

**Atypical Mass:** (A) Sagittal and (B) **o**blique axial T2W MR image**s** show a well-defined intra**-**myometrial lesion (arrow**s**) of intermediate to high signal intensity with non-nodular borders. On the fat-suppressed contrast-enhanced T1W image (C) the lesion show**s avid enhancement and** areas of necrosis (**arrow**). **Oblique-axial** diffusion-weighted image (b=800) **(D)** show**s** high signal intensity (arrow) and restriction on the corresponding ADC map **(E)** (**arrow**), with a measured ADC of 1.25**. These findings** are in keeping with an atypical leiomyoma without 3 or more features suspicious for **leiomyosarcoma and consistent with a cellular leiomyoma, which was histologically proven.**

**Template:**

**Uterus:**

**Orientation:** Anteverted-retroflexed uterus

**Size/Volume:** 10x7x6 cm/ 218cc

**Endometrial thickness:** 5mm

**Junctional zone thickness:** 4mm

**Leiomyoma (we describe the larger and atypical one):**

**Size/Volume**: 7x6x6cm/131cc

**Location:** Intramural – type 3

**Size of stalk if pedunculated:** NA

**Signs and type of degeneration:** NA

**Enhancement characteristics:** Avid enhancement with areas of necrosis

**Differential diagnosis:** signs of atypia

**Features suspicious of sarcomas**

**- Heterogeneity and T2 dark area:** No

**- Flow voids:** No

**- Intra-lesional hemorrhage:** No (not shown on these images)

**- Necrosis area:** Yes

**- Extension to other organ:** No

NA – not applicable
